# Exploring the role of infected keratinocytes during rabies virus infection

**DOI:** 10.1038/s44298-025-00134-9

**Published:** 2025-06-26

**Authors:** Keshia Kroh, Redwan Rahmat, Lineke Begeman, Lars W. van Greuningen, Debby Schipper, Matthijs F. Ravensberg, Stefan Finke, Claude Sabeta, Thijs Kuiken, Corine H. GeurtsvanKessel, Carmen W. E. Embregts

**Affiliations:** 1 Department of Viroscience, Erasmus Medical Center, Rotterdam, the Netherlands; 2 Veterinarian Private Sector, Port Elizabeth, South Africa; 3 Institute of Molecular Virology and Cell Biology, Friedrich-Loeffler-Institut, Greifswald, Germany; 4 Department of Veterinary Tropical Diseases, Faculty of Veterinary Science, University of Pretoria, Onderstepoort, Pretoria, Republic of South Africa

**Keywords:** Virology, Viral pathogenesis, Viral transmission

## Abstract

Rabies virus (RABV), a lyssavirus causing fatal encephalitis, is primarily transmitted via dog bites, though superficial exposures such as scratches or bat bites can also result in infection. The mechanisms underlying transmission through these minor exposures remain unclear. This study investigated the involvement of epidermal keratinocytes in RABV entry. RABV antigen was detected in keratinocytes at inoculation sites in experimentally infected mice and at potential viral entry sites in naturally infected dogs. However, keratinocyte infection could not be replicated in ex vivo skin biopsies from mice or dogs, nor was antigen detected in pre-clinical skin samples. Furthermore, superficial exposure via the inner ear skin of mice did not result in infection. Thus, it remains unclear whether keratinocytes are initially infected or become infected later due to centrifugal spread of RABV. Nonetheless, our findings highlight the need to better understand keratinocyte involvement, especially in superficial RABV exposure.

## Introduction

Rabies is a neglected zoonotic disease that causes at least 59,000 human fatalities per year, of which most cases are reported in Asia and Africa^1^. The disease is characterized by a lethal viral encephalitis that is caused by rabies virus (RABV) or a related lyssavirus. Lyssaviruses are transmitted via the saliva of infected animals, and 99% of the human cases are caused by bites of rabid dogs^2^. Onset of disease can be prevented by rapid post-exposure prophylaxis (PEP), consisting of vaccination and local wound treatment with anti-rabies immunoglobulins (RIG) in case the affected person was not previously vaccinated. While proper PEP can save lives, vaccines and especially RIG are often not available in rabies-endemic countries. Alarmingly, it is estimated that only 1–2% of exposed people in rabies-endemic countries have access to RIG^3,4^. Delayed administration of PEP to a person bitten by a rabid animal allows virus particles from the saliva to infect peripheral nerves, such as those present at neuromuscular junctions. From this site the virus can spread retrograde via the axons to the spinal cord and will finally reach the brain where massive virus replication will occur, leading to the onset of neurological symptoms^5,6^. Incubation times until the onset of clinical disease can range from a few weeks to several years^6,7^. There is currently no treatment against rabies once clinical signs have developed and patients will succumb to the disease within an average of 5–11 days after the onset of neurological symptoms^7^. Given this, rabies has a case-fatality rate that approaches 100%, making it the deadliest infectious disease worldwide.

Most rabies cases are caused by dog bites, and a smaller proportion of cases are caused by other mesocarnivores including foxes, cats and raccoons. These animals all have long and sharp canine teeth, causing deep bites, typically with virus deposition deep in the muscle tissue. Besides these deep bites, also superficial bites and scratches of infected animals can cause rabies, for example by bat bites or scratches of animals that have contaminated saliva on their nails. Several human rabies deaths yearly are caused by bites of vampire bats, which has been of particular concern in Latin America^8^. These exposures result in virus deposition in the skin instead of in the muscle and can easily go unnoticed^5,9^ or, concerningly, do not receive proper PEP given that the wounds are not severe and the risk of RABV infection is often perceived as low. Clinical rabies cases without a clear bite history have been documented and include reports of scratches of a cat^10^, dogs^11^, bats^12^, or wolf^13^, exposure to saliva of an infected animal on scratched skin^13,14^, or even no known animal exposure^15^. These cases all have in common that nerve endings in the skin, and not in muscles, are most likely the route of entry for the virus in the nervous system.

Although the most important cell type to become infected in the pathogenesis of rabies is assumed to be the neuron, it is not clear whether it is the only possible primary cell type to be infected. Infection of non-neuronal cells has been described throughout the infection cycle—infected muscle cells have been observed at the virus entry site^16–18^, astrocytes in the CNS and Schwann cells in the periphery^19,20^, infected epithelial cells^21–23^ and salivary gland acinar cells^22,24^ have been described in later stages of the disease. While few reports show the presence of infected cells in the epidermal skin layer of rabies-infected animals, the role of infected keratinocytes during RABV infection remains unclear. More specifically, it is currently unknown if keratinocytes are the primary infected cell type, and if RABV can spread from keratinocytes to nerve endings located in the skin, leading to spread of RABV to the central nervous system (CNS) and onset of disease.

To explore the role of keratinocytes in the pathogenesis of rabies, we determined whether RABV was present in keratinocytes of experimentally inoculated mice, naturally infected dogs, and ex vivo-inoculated skin biopsies. Keratinocytes were found to be infected at the endpoint of the disease in mice and were not observed in animals that did not develop rabies, nor at pre-clinical time points, or in non-inoculated muscles or footpads. Infected keratinocytes were also observed in skin biopsies of potential bite exposure sites of dogs that were naturally infected with RABV, confirming that the observed keratinocytes in the mice studies were not artefacts caused by the experimental infection. However, the infection of keratinocytes could not be repeated ex vivo. Furthermore, infection of mice via inoculation of the intact, brushed or superficially scratched skin did not result in development of rabies, and no infected skin cells were observed in any of the skin-inoculated animals. Based on our experiments, we confirmed that keratinocytes in both mice and dogs are susceptible to rabies virus infection in vivo. However, observed keratinocyte infection might represent a late rabies phase infection, where RABV spreads from infected CNS to all kind of innervated tissues, including skin.

## Results

### Infected keratinocytes are observed in experimentally and naturally infected animals

**Fig. 1:**
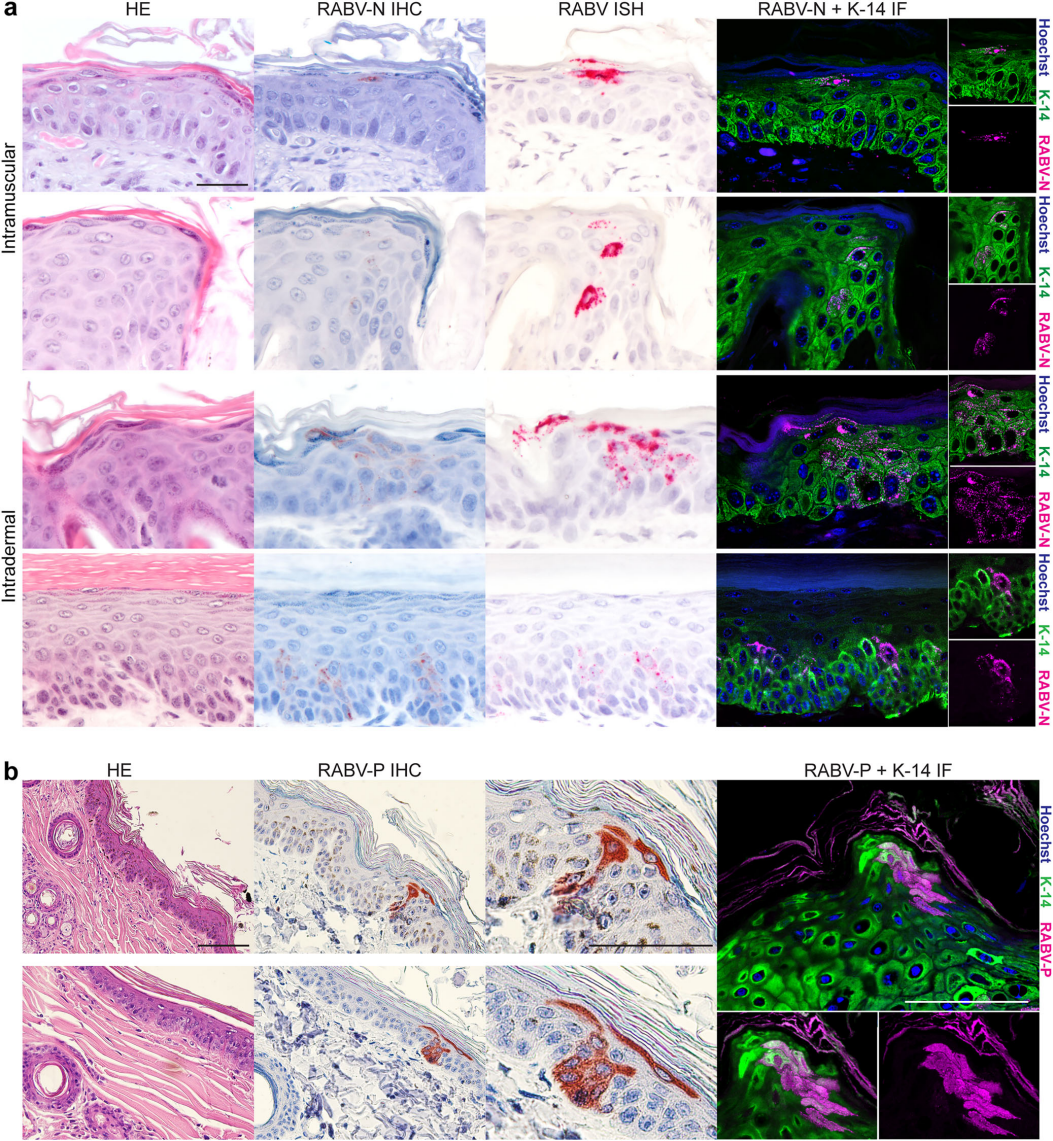
Characterization of RABV-positive skin cells at the intramuscular or intradermal inoculation site of experimentally inoculated mice and in bite/scratch marks of naturally infected dogs. Tissues of mice (**a**) and dogs (**b**) were investigated for general morphology by hematoxylin and eosin (H&E) staining, for the presence of viral proteins by immunohistochemistry (IHC, red) and immunofluorescence (IF, pink), and for the presence of viral RNA by in situ hybridization (ISH, pink). An antibody against keratin-14 (K-14) was used to identify keratinocytes in IHC and IF, and Hoechst was used to stain the cell nuclei in IF. Scale bars represent 50 µm for panel (**a**) and 100 µm for panel (**b**).

Both intradermal and intramuscular inoculation of RABV led to the onset of disease in mice —the highest dose led to an earlier onset of disease and an earlier timing of reaching humane endpoint. In total, 10/18 of the intramuscular inoculated mice and 16/17 of the intradermal inoculated mice developed clinical symptoms and reached endpoint (Supplementary Fig. 1). Immunohistochemical staining of the inoculation site revealed the presence of cells expressing RABV antigen in the epidermal layer of the skin in the intramuscularly inoculated animals (4/18 animals), and in the epidermal layer of the footpad in the intradermally infected animals (4/17 animals) (Fig. 1a, Supplementary Fig. 1). We interpreted that the cells expressing rabies virus antigen were infected with rabies virus. RABV-positive cells were only observed in animals that did develop clinical disease, and were not detected in the contralateral (non-inoculated) leg or footpad or at pre-clinical time points (Supplementary Fig. 2). RABV-positive cells were detected in mice inoculated with the intermediate (1 × 10^4^ TCID_50_, 2/6 of the i.m. inoculated group) and highest (1 × 10^6^ TCID_50_, 2/6 of the i.m. inoculated group, 4/6 of the i.d. inoculated group) inoculation doses (Supplementary Fig. 1). The presence of RABV antigen in the brain was confirmed by immunohistochemistry staining for all animals. Throughout the skin tissue we observed multiple foci of infected cells, which ranged from few RABV-positive cells up to groups of approximately fifteen RABV-positive cells. Most of the RABV-positive cells were observed in the top few layers of epidermal cells, however, especially for the intradermally inoculated animals, foci were also observed in lower epidermal cell layers (Fig. 1a). In none of the tissues we observed RABV-positive skin cells outside of the epidermal layer. The cells expressing RABV antigen in both intradermally and intramuscularly inoculated animals were identified as keratinocytes based both on their location in the epidermis and their morphological characteristics; this was confirmed by immunofluorescent staining with an antibody against keratin-14. In situ hybridization using a probe designed against SHBRV further confirmed the presence of viral RNA in the skin.

Rabies-suspected dogs were euthanized and were examined for bite or scratch marks. During the study period 26 rabies-positive dogs were included, of which seven dogs showed clear marks that could be the possible virus entry site. All of these dogs were confirmed rabies-positive by PCR and positive rabies antigen staining in the brain. Immunohistochemistry revealed that skin biopsies of three out of seven dogs with clear bite marks showed infection of cells in the epidermal layer of the skin. RABV-positive dog skin biopsies all showed foci of approximately ten RABV-positive cells, that were present in the top epidermal cell layers but also extended to lower cell layers (Fig. 1b). Immunofluorescent staining confirmed that, similar to the infection pattern observed in the experimentally infected mice, dogs showed infection of keratinocytes.

### Inoculation on the surface of intact or damaged skin does not lead to infection of keratinocytes or the development of rabies

**Fig. 2:**
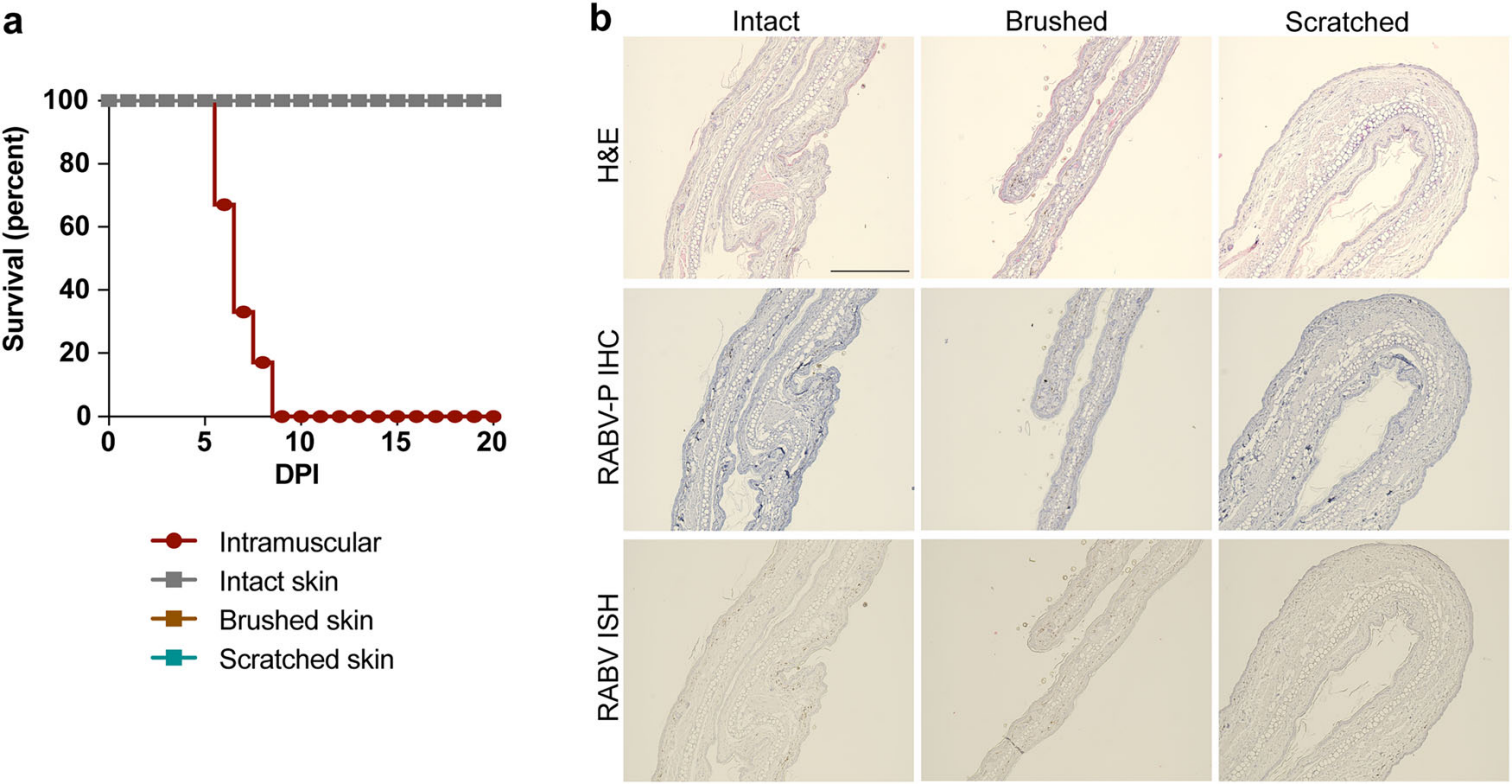
Survival curve and histological examination of the inoculated ears after RABV inoculation on intact, brushed or scratched skin. Mice were superficially inoculated with RABV on the inner ear with the indicated method or injected intramuscularly. Survival was determined (**a**), as well as presence of viral antigen and RNA in the inoculated ear skin (**b**). Scale bar represents 100 µm.

Infection of keratinocytes or other skin cells could not be repeated by ex vivo inoculation of intact, scratched or injected skin biopsies of mice (Supplementary Fig. 3a) and dog skin (Supplementary Fig. 3b), as no RABV-positive cells were observed at any of the included time points. Next, an in vivo experiment was set up to investigate if inoculation of intact, brushed or scratched skin can lead to the infection of keratinocytes and/or the development of rabies. While the animals in the intramuscular inoculated (positive infection control) group all developed neurological signs between day six and eight days after inoculation, no neurological signs or behavioral changes were observed in any of the supradermally (on the surface of the skin) inoculated animals (Fig. 2a). Ears and brains were collected at the end of the experiment and were examined for the presence of viral proteins and RNA by immunohistochemistry and in situ hybridization, respectively. Extensive infection was observed in the brains of the intramuscularly inoculated mice, but no infected cells were found in the ears or brains of the supradermally inoculated mice (Fig. 2b). The lack of neurological signs and the absence of viral proteins and RNA suggests that inoculation via the skin was unsuccessful or was already cleared at day 20 post-inoculation. In the latter situation, the possible infection of keratinocytes did not lead to the induction of rabies.

## Discussion

While almost all reported human rabies cases are caused by dog bites, typically penetrating the muscle layer, less rabies cases are described that are caused by superficial scratches or bites. In a study of 10,971 rabies cases in China, 92% were caused by bites, and 85% were caused by dogs, indicating that 8% (887 cases) are caused by non-bite exposures^25^. In countries with low rabies prevalence and good surveillance, such as the US or Canada, rabies cases from superficial exposures are reported more often, and are almost exclusively associated with bats^5,6,9,26–28^. An analysis of 41 bat-related human rabies cases in the United States showed that only 17% (7 cases) reported a bat bite, while the rest was attributed to more superficial exposures or unknown^29^. In rabies-endemic countries, infections are often not diagnosed due to a combination of ethical, religious, and logistical barriers, including a lack of available diagnostic laboratories and reagents, and lack of knowledge or financial resources^1,6,30^. Superficial bites or scratches may easily go unnoticed, and rabies is often overlooked as a differential diagnosis without evident history of dog- or animal bites. Thus, rabies cases from superficial exposures are likely to be severely underestimated.

Superficially inoculated RABV may use different routes to the CNS than deep penetrating virus, possibly through somatic sensory nerves rather than motor neurons^5,31,32^. We hypothesize that infection of skin cells, such as keratinocytes, might contribute to CNS entry through sensory neurons in cases of superficial exposure.

To our knowledge, the presence of viral antigen in the skin at the initial site of exposure has not been studied, making it unclear if keratinocyte infection contributes to viral CNS entry and rabies development. High nerve density in the skin and intimate contact of intra-epidermal nerve endings with keratinocytes^33,34^ suggest possible virus transfer. Moreover, keratinocytes could play a key role in immune functions and pathogen defense^35,36^, similar to herpes simplex virus-1 (HSV-1) infections, where keratinocytes have been shown to transmit the virus to sensory nerves^37,38^ and release cytokines and recruit immune cells to counteract viral propagation^37,39,40^.

RABV antigen presence in the skin in the clinical phase of rabies has been described before and is used for diagnostic purposes^21,41,42^. Diagnostic samples are usually taken at locations proximal to the brain, such as the neck or muzzle, where RABV particles are often detected in parafollicular nerve fibers as a consequence of the centrifugal spread from the CNS to the periphery^21,41–47^. Few reports document the presence of RABV antigen in non-nervous cell types of the skin and include epithelial cells in experimentally infected hamsters^48^, skunks^22^, rabid dogs^23^, and human nuchal skin^21^, indicating antigen positivity might not be restricted to the parafollicular nerve fibers.

In the present study, we observed infected keratinocytes at the inoculation site of i.m. and i.d. inoculated mice at the end-stage of infection. Infected keratinocytes were only found in animals that developed disease and found more often in animals inoculated with higher viral doses. This suggests a dose-dependent effect. Although infected keratinocytes were only observed in a fraction of the animals, only a relatively small area was investigated by immunohistochemistry. The few infected cells or cell patches that we observed can be easily missed if only few thin slides are prepared per tissue, so the observed incidence of infected keratinocytes might be an underestimation. No infection was observed besides keratinocytes in the epidermal layer, suggesting that the virus did not spread to other cell types, either in the epidermis or in the adjacent dermis. Infected keratinocytes were also observed in skin biopsies of bite or scratch sites of rabies-diagnosed dogs, representing possible viral entry sites. This confirms that the observation of infected keratinocytes also occurs in natural infections and in natural hosts. Like the observation in mice, keratinocytes were the only infected cells in the skin.

The infection of keratinocytes observed in our experiments could have promoted an early and efficient virus replication, contributing to virus dissemination to the CNS and onset of disease. A virus dose that is too low to result in infection by itself might reach levels sufficient to trigger infection when amplified through replication in keratinocytes. On the other hand, the infection of keratinocytes might be a consequence of centrifugal spread of the virus from the CNS in later stages of the infection through viral release at nerve endings^20^. We believe this to be more likely, since we did not observe any infection of keratinocytes in mice sacrificed in the pre-clinical stage of rabies. Since there is a direct contact (reflex loop) in the spinal cord between the somatic sensory and somatic motor neurons innervating the site of inoculation^5^, RABV might even spread from the muscular site of inoculation to the adjacent epidermis via the neuronal route, without having to replicate in the brain first^5,7^. In that case, RABV antigen would be expected in the contralateral (non-inoculated) leg or footpad as well, but was not observed in our experiments. Since it is practically impossible to trace back the exact contralateral innervation site, infected cells could have been missed. In addition, the virus might still be present in peripheral innervating nerves^20,49^. Nevertheless, the observation that infected keratinocytes were exclusively found in inoculated tissues suggests that there may be another contributing factor to their presence besides only the centrifugal spread to peripheral tissues in end stages of disease.

We could not confirm the infection of keratinocytes by ex vivo inoculation of skin biopsies of mice and dogs, further suggesting that keratinocytes might not be susceptible to infection via this route. However, ex vivo studies can be challenging in general due to rapid tissue degradation, which limits the ability of observing viral infection. Ultimately, the risk of developing rabies through superficial virus exposure and the role of local viral replication in keratinocytes is best studied in in vivo experiments.

The inoculation of mice on the inner skin of the ear did not result in the onset of disease, any abnormal behavior, or weight loss. C57BL/6 mice are susceptible to infection with RABV, and the i.m. inoculated group developed rabies within six to eight days post-inoculation. No viral antigen was detected in the brains, salivary glands, tongue, or nuchal skin biopsies in any of the skin-inoculated animals. We chose the ear as site of superficial RABV exposure, since it is densely innervated, lacks hair, and the nerves connecting CNS to ear are short^50^. Possible reasons for the failure of inducing rabies through supradermal inoculation are: the volume of inoculum was too small or did not stay concentrated long enough on the applied skin area; the experimental method inadequately replicates superficial biting or scratching by a rabid animal; or this is not a viable route of infection. While we applied the inoculum supradermally, intra-epidermal inoculation might represent an actual bat bite more adequately, depositing the virus deeper and more concentrated, increasing the chance of viral entry into nerve endings. For the present study, we decided against this approach, as our aim was to investigate the possibility of RABV infection through superficial exposure via a potential infection of keratinocytes.

In our study, we observed the presence of RABV antigen in epidermal keratinocytes at the site of exposure in both naturally and experimentally infected rabid animals. None of the mice that were superficially exposed to RABV through the ear skin developed rabies. Therefore, we propose that the possibility of keratinocyte infection is low, especially through superficial (apical) application. Further studies are needed to determine whether RABV reaches the keratinocytes through centrifugal spread at later stages of the disease, or if the infection of keratinocytes occurs already at the time of exposure. If the latter, it would be important to determine whether local virus amplification in keratinocytes as a primary site of infection increases the chance of virus spread to the CNS and initiation of clinical disease. Contracting rabies through superficial skin exposure might be rare, but it should not be ruled out.

## Materials and methods

### Animal studies

Experiment 1 was performed primarily with the goal of optimizing the intramuscular and intradermal RABV inoculation models, and skin sections from RABV-inoculated mice were examined retrospectively for the presence of infected keratinocytes. Experiment 2 was performed to investigate the pre-clinical immune response to RABV, and skin samples were investigated retrospectively. Experiment 3 was performed to answer the question whether keratinocytes could be infected by contact of virus with intact or damaged epidermis, and whether such infection would lead to clinical disease. In all three experiments, inoculation was performed under general anesthesia via isoflurane inhalation. Euthanasia, upon showing neurological signs or at the end of the experiment, was performed by cardiac puncture under isoflurane inhalation anesthesia. In all three experiments, the inoculated groups were directly compared to each other, and weight, behavior and mortality (e.g. euthanasia upon showing neurological signs) was recorded at least daily (units: single animals). No uninfected groups were included to limit the number of experimental animals. Six animals were included in every group; this number was chosen based on experience and practical reasons as animals were housed in groups of three in type-I rodent cages. Given the descriptive nature of our studies, and animal welfare reasons, we decided on a low number of animals per group. Groups were divided randomly; however, litters were not mixed due to welfare reasons. Blinding was not performed, as daily welfare assessments included inspection of the inoculation site, and early detection of onset of neurological signs was essential to ensure animal welfare. No animals were excluded throughout the studies – none displayed the a priori set exclusion reasons (severe (local) effects of the inoculation or unrelated health issues). Confounders related to treatment/measurement/cage order were not controlled as they were previously not observed. The animal experiments were performed under a project license (2019-0060) approved by the competent Dutch authority and in compliance with Dutch regulation for the protection of animals used for scientific purposes (implementing EU directive 2010/63) and other relevant regulations. The specific study protocols (18-7204-01 for Experiment 1, SP23000255 and SP2300049 for Experiment 2, and SP2200149 for Experiment 3) were approved by the institutional Animal Welfare Body.

Experiment 1 – intramuscular and intradermal inoculation: Mice (wild-type C57BL/6, female and male (50:50), six-to-eight-week-old, six animals per group; 36 animals in total) were inoculated with 10^2^, 10^4^ or 10^6^ TCID_50_ (intramuscular inoculation, quadriceps femoris) or 10^4^, 10^5^ or 10^6^ TCID_50_ (intradermal inoculation, footpad) of silver-haired bat rabies virus (SHBRV). The strain was isolated from the brain of a human rabies patient and is commonly used to study the pathogenesis of wild-type (street) rabies viruses^51–53^. The virus was propagated in the human neuroblastoma cell line SK-N-SH, as described previously^54^. Briefly, SK-N-SH cells were inoculated in suspension with SHBRV at an MOI of 0.01 for 1 h, after which they were transferred to cell culture flasks. Virus was harvested from the supernatant 3 days post inoculation. A low number passage (p3) was used for inoculation of mice and stocks were sequenced on Illumina MiSeq flow cells using targeted enrichment by VirCapSeq^55,56^ to rule out the appearance of culture adaptations. Inoculation was done by a single intramuscular injection on day 0 of the experiment (after a seven-day acclimatization period) in the left hind leg or by intradermal injection in the footpad of the left hind leg. For both inoculation routes a volume of 25 µl was used. Mice were monitored daily and were euthanized upon showing neurological signs or at the end of the experiment (day fifteen post-inoculation).

Experiment 2 – pre-clinical investigations: Mice (wild-type C57BL/6, male, six-to-eight-week-old, six animals per group, twelve animals in total) were inoculated with 10^5^ TCID_50_ SHBRV in 25 µl by a single intramuscular inoculation on day 0 of the experiment (after a seven-day acclimatization period), as this dose was found optimal to induce consistent clinical disease in mice in experiment 1. Mice were monitored daily and were euthanized at an early (two days post inoculation (dpi)) and late pre-clinical (five dpi) time point.

Experiment 3 – inoculation on intact, brushed or scratched skin: Mice (wild-type C57BL/6, male, six-to-eight-week-old, six mice per group, 24 animals in total) were inoculated with SHBRV on the skin of the left ear. The ear was chosen as the inoculation site as it does not require the removal of hair, which potentially damages the skin, and because of its high innervation level. Ears were inoculated with a cotton swab, and a final viral dose of 10^5^ TCID_50_ in 5 µl per animal was used for inoculation. The inoculation procedure was optimized by weighing swabs containing plain culture medium (DMEM) before and after swabbing ears of mice (obtained from other experiments, see ex vivo section below)—the determined volume that remained on the skin after wiping the swab on the ear was used to dilute the virus stock accordingly. Ears of RABV-inoculated mice were either untreated (intact), brushed with a toothbrush (three strokes per ear), or scratched (three evenly spaced parallel scratches per ear) with a needle. Inoculation of the skin was performed directly after, by wiping the virus-containing swab on the inner part of the ear. The scratching was optimized in a way that resulted in damage to the epidermis only, which was verified by Hematoxylin and Eosin (H&E) staining of pieces of skin (obtained from other experiments, see ex vivo section below). To prevent mice from cleaning their ears, which could lead to ingestion of the virus, an Elizabethan collar (Lomir) was placed for eight hours after inoculation. Intramuscular inoculation with 10^5^ TCID_50_ (25 µl) in the hind leg was included as positive control for infection. Mice were monitored daily and were euthanized upon showing neurological signs, or at day 21 post-inoculation.

### Skin collection from rabies-infected dogs

Skin biopsies were collected from rabies-suspected dogs in the Nelson Mandela Bay Municipality, Eastern Cape, South Africa, during an ongoing rabies outbreak in 2022^57^. Dogs with a suspicion of rabies, based on aggressive or abnormal behavior or neurological signs, were euthanized and a brain sample was taken using the straw method^58^ for rabies diagnostics by PCR^59^. In addition, the presence of RABV antigen in the brain was determined by immunohistochemistry staining as described below. Dogs were visually inspected for bite or scratch marks and, if present, a piece of 1 ×1 cm skin was collected from every clear mark that represented a potential viral entry site. All bite marks showed advanced signs of healing and scar tissue formation, and were estimated to be 2–5 weeks old. Marks were found in seven (six males and one female, four strays and three owned dogs, average age of 3.5 years (range 1–10)) out of 26 rabies-positive dogs. Skin biopsies were fixed in formalin and were processed for immunohistochemistry and immunofluorescence as described below. Dog examination, euthanasia and sampling was performed by trained veterinarians and skin samples were taken under the ethical permit Section 20 from the Agriculture, Land reform and Rural development Department of Republic of South Africa (Ref 12/11/1/1(a) (2748KL).

### Ex vivo infections

Skin was collected from the back area of mice (*n* = 2) and from the belly and neck (shaved) area of dogs (mixed breed, *n* = 2). All skin specimens were post-mortem rest materials from other animal experiments and were taken after the initial experiments had finished and the animals were euthanized. The animal experiments were performed under project licenses (mice: 2019-0060, dogs: AVD11500202010905) approved by the competent Dutch authority and in compliance with Dutch regulation for the protection of animals used for scientific purposes (implementing EU directive 2010/63) and other relevant regulations. Three skin biopsies per animal and anatomical site were collected. Skin pieces were rinsed in cold phosphate-buffered saline (PBS), after which 8 mm diameter biopsies were collected with a biopsy punch. Biopsies were placed in 24-well plates in ex vivo culture medium (DMEM mixed with Ham’s F12 (1:1) supplemented with 2% (v/v) FBS, penicillin, streptomycin, L-glutamine and amphotericin B). Biopsies were exposed to SHBRV at a final concentration of 10^6^ TCID_50_ (in 25 µl) by three different routes: by pipetting the virus on the intact dry surface, by first scratching the skin (three evenly spaced parallel scratches) superficially with a needle followed by pipetting of virus on the skin, or by injecting the virus intradermally. Tissue biopsies were incubated with the virus for one hour. To isolate virus infection via the epidermis, as opposed to the dermis through virus presence in the culture medium, the biopsies were then rinsed with ex vivo culture medium three times, and placed in fresh ex vivo culture medium. Virus-exposed tissue biopsies were removed at 24- and 48-h post-inoculation and at five days post-inoculation. Tissue biopsies were fixed in 10% neutral-buffered formalin and were processed for immunohistochemistry as described below.

### Immunohistochemistry and immunofluorescence

Formalin-fixed tissues were paraffin-embedded, and formalin-fixed mouse feet were decalcified with 10% EDTA for ten days before embedding. Presence of viral proteins was investigated by immunohistochemistry (IHC) and immunofluorescence (IF) on 3-µm-thick slides, in accordance with standard practice for rabies diagnostics^23,56,58^. Antigen retrieval was performed by boiling the slides for fifteen minutes in citric acid buffer, and endogenous peroxidase activity was blocked by incubation for ten minutes in 3% hydrogen peroxide in PBS. The RABV nucleoprotein (RABV-N) and phosphoprotein (RABV-P) were detected using the 5DF12 antibody^60^ and the P160-5 antibody (kindly provided by S. Finke^61^) as primary antibodies, respectively. Isotype controls were taken along for each staining and were found to be consistently negative. Horse-radish peroxidase (HRP) secondary antibodies were used in IHC and revelation of HRP was performed by 3-Amino-9-ethylcarbazole (AEC). Brain tissue of RABV-infected mice was taken along as positive control for primary antibody binding and HRP revelation. Keratinocytes were stained using a Keratin (K-)14 antibody (Biolegend, clones Poly19053 and Poly9060). Hoechst was included in the IF staining for visualization of the nuclei. Following antigen retrieval, slides were incubated with the primary antibodies for 1 h at room temperature. After washing, slides were incubated with fluorescently labeled secondary antibodies (goat-anti-chicken Alexa Fluor (AF) 647, goat-anti-rabbit AF488, goat-anti-mouse AF647, all Invitrogen), and Hoechst (Invitrogen) for 1 h at room temperature. Slides were examined using an Olympus BX51 microscope (IHC) or a Zeiss LSM700 confocal microscope (IF).

### In situ hybridization

To provide evidence of RABV infection of IHC-positive keratinocytes by another method^62,63^, we examined tissue sections (5-µm thick) for the presence of rabies virus RNA by use of in situ hybridization (ISH) using the commercial RNAScope 2.5 FFPE Assay (Advanced Cell Diagnostics) and a probe specifically designed to target the SHBRV glycoprotein gene (GenBank AY705373.1). The manufacturer’s instructions were followed and included deparaffinization, dehydration, and pre-treatment of the tissue sections to make the target RNA accessible. The probe was hybridized at 40 °C for two hours, after which the signal was amplified with six amplification steps before the signal was visualized with Fast Red. Positive (UBC, targeting a mouse housekeeping gene) and negative control (DapB, targeting an unrelated bacterial gene) probes were taken along. Brain slides of experimentally inoculated mice, with PCR-confirmed positivity in brain, and non-inoculated mice, were taken along as additional positive and negative controls. The slides were counterstained with hematoxylin and mounted with EcoMount. Slides were examined using an Olympus BX51 microscope.

## Supplementary information

Supplementary figures

Author Checklist ARRIVE E10

## Data Availability

The datasets used in the current study are provided within the manuscript and supplementary information files or are available from the corresponding author upon reasonable request.

## Code availability

No custom code or scripts were used to generate or analyze datasets.
