# Supplementary material for: Exploring the role of infected keratinocytes during rabies virus infection
Source: Npj Viruses. 2025 Jun 26;3:53. doi: 10.1038/s44298-025-00134-9 (PMC12202707; doi:10.1038/s44298-025-00134-9)
Supplement: Supplementary file 1 — Supplementary figures [file 44298_2025_134_MOESM1_ESM.pdf]

Supplementary figures

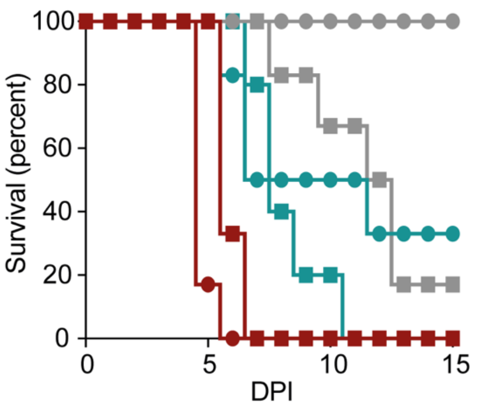

Inoculation Route	Dose ($TCID_{50}$)	$RABV^+$ keratinocytes
I.D.	1x10^4	0/5
I.D.	1x10^5	0/6
I.D.	1x10^6	4/6
I.M.	1x10^2	0/0
I.M.	1x10^4	2/4
I.M.	1x10^6	2/6

Supplementary figure 1. Survival curve of mice inoculated with various doses of SHBRV by intradermal (I.D.) or intramuscular (I.M.) inoculation, and the incidence of $RABV^+$ keratinocytes in skin biopsies of animals that developed disease. Six animals were inoculated per group, table numbers indicate the numbers of animals within these groups that developed disease.

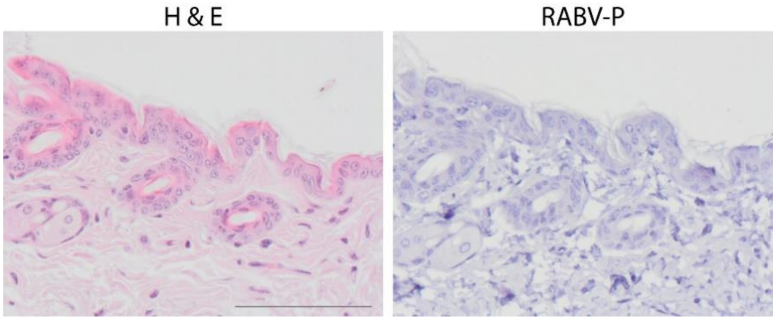

Supplementary figure 2. Morphological (H&E) and immunohistochemical (IHC) examination of murine skin taken in pre-clinical phase of experimental RABV infection. Representative images of skin sections taken from mice sacrificed two days after i.m. inoculation. Stained with H&E and antibody targeting the RABV-P protein. Scale bar represents 100 µm.

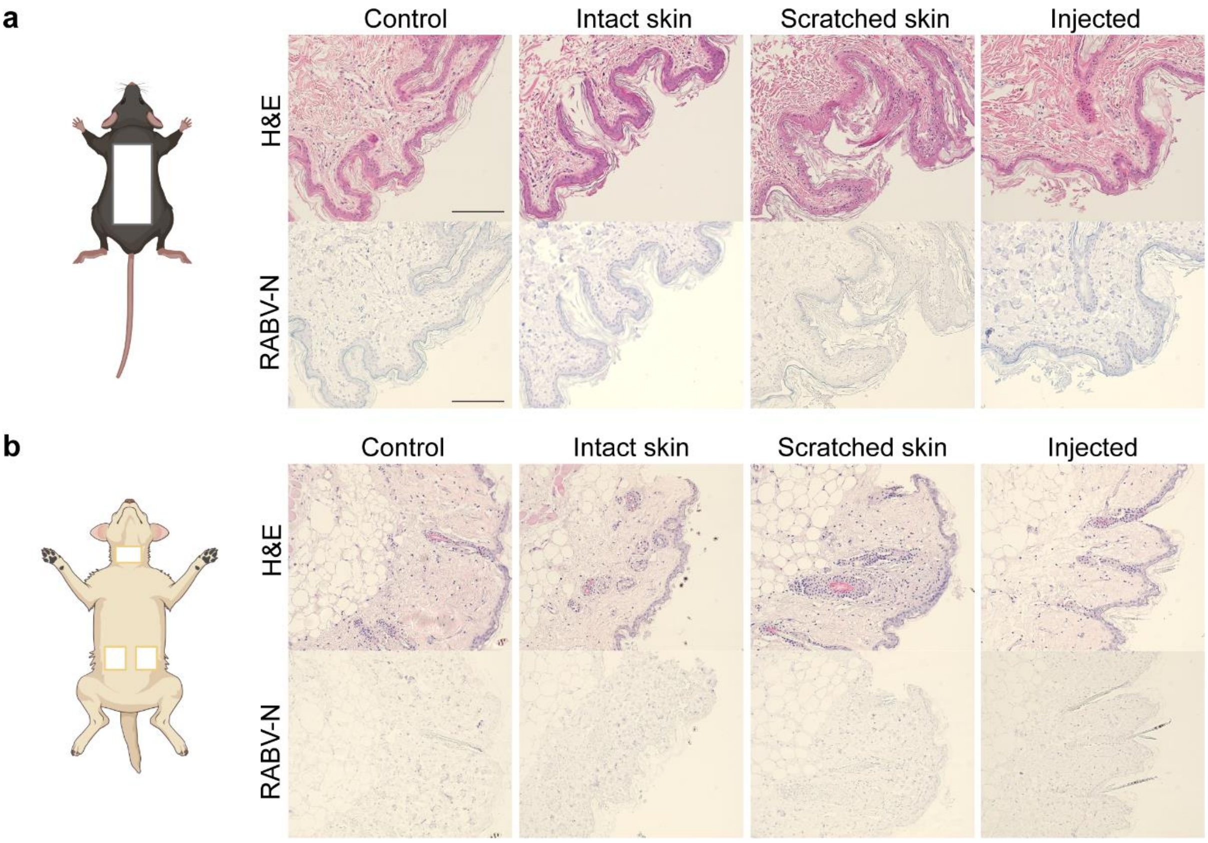

Supplementary figure 3. Morphological (H&E) and immunohistochemical (IHC) analysis of *ex vivo* inoculated mice (a) and dog (b) skin biopsies (Created by BioRender). Anatomical sites of skin biopsies are shown on the left, representative images of skin biopsies fixed after 48 hours post-inoculation and stained with H&E and an antibody targeting the RABV-N protein are shown in the right panel. Scale bar represents 100 µm.
